# Unveiling the Devastating Impact of Atypical and Rapidly Evolving Guillain-Barre Syndrome: A Report of Two Cases

**DOI:** 10.7759/cureus.71057

**Published:** 2024-10-08

**Authors:** Siddhi Gawhale, Sampada Tambolkar, Manojkumar G Patil, Poornima Vonteru, Shailaja Mane

**Affiliations:** 1 Pediatrics, Dr. D. Y. Patil Medical College, Hospital and Research Center, Dr. D. Y. Patil Vidyapeeth (Deemed to be University), Pune, IND; 2 Pediatric Neurology, Dr. D. Y. Patil Medical College, Hospital and Research Center, Dr. D. Y. Patil Vidyapeeth (Deemed to be University), Pune, IND

**Keywords:** atypical guillain-barré syndrome, descending weakness, immune-mediated, immunomodulation, multidisciplinary approach

## Abstract

Guillain-Barré syndrome (GBS) is an immune-mediated demyelinating disease; weakness is typically ascending in nature with areflexia. Any deviation from this typical course becomes a diagnostic challenge with variable prognosis. Here, we report cases of GBS presenting with rapidly evolving descending weakness with predominant bulbar involvement and dysautonomia as an atypical presentation, which can lead to delay in diagnosis and treatment. We also highlight how atypical GBS are slow responders to immunomodulation therapy like intravenous immunoglobulin (IVIG) and plasma exchange, and hence, a multidisciplinary approach would modify the outcome.

## Introduction

Guillain-Barré syndrome (GBS) is a progressive immune-mediated polyneuropathy with a variety of clinical manifestations. "Ascending paralysis" is a characteristic that most frequently manifests [1]. In 1859, Octave Landry wrote about an illness that progressed to ascending paralysis, respiratory failure, and eventually death [2]. It was identified as syndrome de radiculonévrite in 1916 by Guillain, Barré, and Stroll, who also documented two cases of severe self-limited motor weakness with depressed reflexes and positive outcomes [3]. GBS is diagnosed based on the patient’s clinical presentation, history, progression, neurological examination, electrophysiological studies, and cerebrospinal fluid (CSF) studies [4]. The three forms of GBS-acute inflammatory demyelinating polyradiculoneuropathy (AIDP), acute motor sensory axonal neuropathy (AMSAN), and acute motor axonal neuropathy (AMAN)-can be distinguished using electrophysiological investigations that indicate peripheral nerve damage [5]. The GBS variation known as AIDP is characterized by lymphocyte, mononuclear, and macrophage infiltration together with segmental demyelination at the proximal nerve segments and nerve roots. While AMAN is more common in younger individuals, AIDP is the most common variant of GBS globally. In AIDP, the immune attack is against the peripheral nerve myelin [6], and decreased compound muscle action potentials (CMAP) is seen in AMAN [7]. This inflammatory demyelinating polyradiculopathy has a few rare variations, including Miller-Fisher syndrome, which is characterized by ophthalmoplegia, ataxia, and areflexia; Bickerstaff's brainstem encephalitis, which is characterized by altered consciousness, ataxia, hyperreflexia, and ophthalmoplegia; acute motor-conduction block neuropathy; and a pharyngeal-cervical-brachial variant that affects the face, neck, and flexor muscles in the upper limbs, ptosis, and spares the lower limbs [8].

The incidence rate of GBS is 0.69 per 100,000 person years, with a peak observed in children aged two. Males had a higher incidence rate, with approximately 63% of cases preceded by infection. A mortality rate of 11.5% was recorded in India. A total of 38.5% of participants in the research reported bulbar weakness, while 19.2% reported involvement of other cranial nerves, with facial nerve being more common. Dysautonomia was seen in 17.3% of cases, and respiratory failure was observed in 28.8%, out of which 19.2% needed mechanical ventilation [9].

We discuss here two cases with descending type of paralysis with severe bulbar involvement presenting within a few hours of initial presentation of weakness with autonomic disturbances. We emphasize the atypical descending presentation with predominant bulbar involvement and importance of early diagnosis and treatment for a better outcome. These patients had poor response to management and had prolonged hospital stays and related complications.

## Case presentation

Case 1

A 13-year-old boy presented to the emergency department complaining of increasing upper extremity weakness, difficulty swallowing, and pooling of saliva with inability to talk, followed by truncal weakness progressing to lower extremities over 12 hours of initial symptom. The patient described his weakness as progressing over time as not being able to carry out his daily activities, with the weakness felt at the back level being defined as difficulty getting up from a lying down position and worsening as difficulty standing and walking, having difficulty in swallowing, speech, and breathing. He did not have numbness, paresthesia, headache, seizures, fever, meningeal signs, visual disturbances, incontinence, or a history of recent vaccination. The weakness was rapidly progressive with simultaneous bulbar involvement. He had a history of bilateral parotitis suggestive of mumps 15 days ago, which settled on supportive therapy not requiring hospitalization. On presentation to the emergency department, he had a paradoxical breathing pattern with a weak gag reflex, pooling of saliva, and an inability to phonate with unimpaired consciousness. For this, he was electively intubated and mechanically ventilated. Bilateral power according to the Medical Research Council scale for muscle power (MRC) was 2/5 in the upper limbs and 4/5 (MRC) in the lower limbs. He had no sensory loss, upper and lower extremities deep tendon reflexes were depressed, and preserved superficial reflexes. Apart from his routine investigations, which were normal, CSF showed albumino-cytologic dissociation. He was evaluated for polyneuropathy secondary to occult diphtheria; the throat swab was negative for Albert stain; and culture was negative. The possibility of mumps polyneuropathy was evaluated by testing his serum and CSF for mumps antibodies; the serum sample was positive for mumps antibodies, whereas the CSF was negative. However, the possibility of post viral polyneuropathy cannot be ruled out. Nerve conduction velocity (NCV) studies showed an absent F wave suggestive of demyelinating polyneuropathy.

Over the course, the patient had worsening upper limb power compared to the lower limb, areflexia, and was eventually quadriplegic. He also had autonomic instability in the form of hypertension and was started on beta-blockers. His hemogram and metabolic profile were within normal limits. The magnetic resonance imaging (MRI) of the brain did not show any obvious findings. He was given intravenous immunoglobulin (IVIG) at 2 g/kg over five days, with other supportive care. However, there was no clinical improvement, and repeat NCV also showed no improvement, so 15 days post-IVIG therapy, plasmapheresis was started, and alternate-day five cycles were done at 50 ml/kg. A tracheostomy was performed, and deep venous thrombosis prophylaxis was also started with physiotherapy and other supportive care. His tracheostomy was eventually decannulated, and with some limb weakness, he was discharged on supportive care.

Case 2

An 11-year-old female presented to the emergency department with an acute onset of all four limbs weakness (upper limb followed by lower limb) and an inability to get up from bed that reached its nadir in less than six hours with difficulty swallowing, drooling of saliva, a change in voice, and difficulty breathing. She had a past history of being diagnosed with GBS and treated with IVIG, not requiring mechanical ventilation at three years of age in a tertiary care center. On examination, she had muscle power of 2 out of 5 (MRC) in all four limbs with preserved but weak deep tendon reflexes. There was predominant bulbar palsy with bilateral lower motor facial nerve palsy and preserved consciousness. The worsening of limb weakness over a few hours was noted, and the patient was completely quadriplegic within the next 10 hours of admission. She eventually developed a labile heart rate and hypertension, suggesting autonomic dysfunction. She eventually also had fever spikes and blood pressure fluctuations, for which a septic workup was sent, and was empirically started on broad spectrum antibiotics and vasoactive drugs. Differential diagnosis of weakness that peaks in less than 12 hours includes periodic paralysis, intoxications, tick palsy, neuroparalytic snake bite, poliomyelitis, and vascular myelopathy were considered. She was mechanically ventilated on admission, and the NCV showed absent F waves, whereas the MRI brain and MRI spine were normal. CSF studies showed raised protein with a cell count of six cells/high-power field done at the second week of suggestive albumino-cytologic dissociation, and CSF viral polymerase chain reaction (PCR) was negative. As in this case, the progression was very rapid; a serum antibody profile was sent, which was positive for the anti-GM1 antibody, suggesting multifocal motor neuropathy GBS. She was treated with IVIG at 2 gram per kg of weight with other supportive care. However, she failed to show any clinical improvement or NCV. She had worsening consciousness on day 4 of her stay, for which a repeat MRI of the brain was performed, which showed diffusion restriction of the bilateral cerebellar hemisphere. Suspecting brainstem encephalitis, she was given a pulse dose of methylprednisolone. However, no improvement was noted and is currently paraplegic and mechanically ventilated and planned for plasmapheresis and tracheostomy, with continuation of other supportive care.

## Discussion

GBS is often clinically diagnosed based on the progression of weakness and antecedent infectious etiology. GBS is characterized by symmetric weakness, progressive nature, absence of deep tendon reflexes, and preservation of consciousness as its diagnostic features. Atypical symptom presentation can complicate diagnosis and potentially delay the initiation of IVIG administration. Additionally, our cases illustrated that atypical presentation and rapid symptom progression were linked to a poor response to treatment and delayed recovery. The strength of these case reports is the distinct clinical presentation with predominant involvement of bulbar muscles requiring immediate mechanical ventilatory support and rapid progression of the course, which made these cases unique and challenging to align with established ascending GBS clinical variants. At the initial presentation, differential diagnoses considered included cerebral ischemic stroke, tumors, diphtheria polyneuropathy, and botulism. Variants of GBS depend on the nerve fibers involved, such as motor, both sensory and motor, cranial, or autonomic [10]. The most common variant involves demyelinating nerve fiber injury, while variants involving axonal motor and axonal sensory fibers have a rapid progression and poor prognosis [11]. Axonal involvement is typically preceded by *Campylobacter jejuni* infection [12]. GBS descending variants encompass the Bickerstaff brainstem encephalitis and Miller-Fisher variant [13].

*Campylobacter jejuni*, *Mycoplasma pneumoniae*, Epstein-Barr virus, hepatitis E virus, and cytomegalovirus have consistently been linked to GBS in case-control studies [14]. In addition to basic hemogram and metabolic profile, CSF analysis reveals albumino-cytologic dissociation due to increased blood-nerve barrier permeability at the proximal nerve root level, observed in 90% of cases within 10-14 days of illness. Electrodiagnostic studies are conducted to assist in diagnosing and evaluating response to treatment, as well as distinguishing between axonal and demyelinating subtypes. Variants of demyelinating conditions are characterized by the early absence of F waves, prolonged latency, and the absence of H reflexes, indicating issues with proximal nerve trunks or roots [15]. The action potential of sensory nerves and compound muscles is reduced in the AMSAN subtype. The sensory ataxic variant and Miller-Fisher variant exhibit reversible neuropathy confined to the sensory nerves, while AMSAN and pharyngeal-cervico-brachial variant affect both sensory-motor nerves [16]. While not commonly used for diagnosis, MRI plays a crucial role in identifying GBS variants such as the Miller-Fisher variant and the pharyngeal-cervico-brachial variant, and it helps rule out conditions that mimic GBS, such as stroke, myelopathy, brainstem infection, or cauda equina syndrome. An MRI of the spine reveals enhancement of intrathecal spinal nerve roots and cauda equina, with an 83% sensitivity [17]. Enhancement of posterior column nerves or cranial nerves is seen in Miller-Fisher syndrome [18]. Supportive care is the cornerstone of treatment. Not all patients need intensive care, but the presence of one or more of dysautonomia, bulbar palsy, rapidly worsening weakness, and evolving respiratory distress needs admission to the intensive care unit (ICU) [19].

The cornerstones of GBS treatment include plasmapheresis and IVIG. IVIG binds to different pathogenic antibodies and increases their clearance by linking to the constant and variable sections of immunoglobulin class G and receptors on macrophages and B cells. It improves CD8+ T-cell activity through an unidentified mechanism [20]. IVIG is less advisable after four weeks and is more effective if administered within two weeks of the onset of symptoms [21]. Complements, cytokines, autoantibodies, immune complexes, and other inflammatory mediators are removed by plasma exchange. In the randomized controlled trials, the average time to improvement varied from six to 27 days. A total of 6% to 16% of patients experience "treatment-related fluctuations (TRIF)," which are defined as one or more bouts of relapse or worsening following an initial improvement in symptoms. Further IVIG or plasma exchange cycles are recommended for these patients. The role of steroids is not clear. The combination of IVIG with methylprednisolone didn’t show any long-term advantage over IVIG alone in one trial [22].

The outcome of GBS is favorable, as about 80% of patients can work independently, and over 50% experience complete recovery within one year [23]. Persistent low-amplitude compound muscle action potential in follow-up studies indicates axonal loss, which signifies a poor prognosis and could lead to residual weakness. Even with an aggressive multidisciplinary approach, residual symptoms like weakness and paresthesia may endure past the one-year mark, impacting the quality of life for patients. In GBS, the mortality rate ranges from 3% to 7%, with the quality of supportive care playing a significant role, and it can reach approximately 20% in patients dependent on mechanical ventilation [24]. An early multidisciplinary approach involving physiotherapy, occupational and speech therapy, dietary support, and family and psychological support is crucial. The superiority of IVIG or plasmapheresis as a treatment option has not been conclusively established. It is more logical to administer IVIG after plasmapheresis, as giving IVIG first would result in the loss of immunoglobulins during apheresis [25]. Therefore, the choice of treatment depends on the progression of the illness, resource availability, and the physician's experience.

## Conclusions

GBS was classically known to have ascending quadriparesis progressing over days to weeks; however, any deviation from this should also raise concern about GBS, and prompt investigation and immunomodulation therapy should be started. Atypical GBS may present as a descending variant, as in our cases, may have asymmetrical paresis, may be regional or localized variants, may have bulbar palsy or brainstem involvement, or may have ophthalmoplegia. These cases also highlight the rapidity of worsening clinical features, their poor response to treatment, requiring prolonged intensive care, and the poor recovery and residual weakness seen in cases with atypical GBS. Hence, more aggressive immunomodulatory therapy and a multidisciplinary approach are the mainstays of therapy in atypical GBS to improve the outcome.
